# Diffusion Tensor Imaging and Advanced Diffusion Imaging in Post-Stroke Aphasia Recovery

**DOI:** 10.3390/tomography12020028

**Published:** 2026-02-23

**Authors:** Irem Yesiloglu, Melissa Stockbridge, Zafer Keser

**Affiliations:** 1 Department of Neurology, Mayo Clinic, Rochester, MN 55902, USA; yesiloglu.ayseirem@mayo.edu; 2 Department of Neurology, Johns Hopkins University, Baltimore, MD 22218, USA

**Keywords:** aphasia, DTI, diffusion imaging, DSI, CSD, DKI

## Abstract

**Background:** Stroke is a leading cause of mortality and long-term disability, and aphasia is among its most common and debilitating sequelae. Diffusion tensor imaging (DTI) and advanced diffusion imaging techniques enable the assessment of white matter integrity and provide clinically relevant measures in post-stroke aphasia. **Methods:** We conducted a comprehensive review of studies applying DTI or advanced diffusion imaging to investigate structural connectivity in adults with post-stroke aphasia (PSA). PubMed, CENTRAL, Ovid MEDLINE, and Embase were searched, and eligible studies were synthesized according to their diagnostic, prognostic, or therapeutic focus. **Results:** Ninety-five studies were included. Of these, 59 were classified as diagnostic, 17 as prognostic, and 19 as therapeutic. Most studies employed conventional DTI (n = 77), while a growing body of research utilized advanced diffusion models, including CSD, DSI, and DKI (n = 18). **Conclusions:** This comprehensive synthesis demonstrates the evolution of diffusion imaging in PSA research. While conventional DTI has provided foundational insights, advanced diffusion methods offer superior characterization of complex fiber architecture and improved clinical–anatomical correlation. Diffusion-derived markers of dorsal and ventral language pathways were consistently associated with language performance, while connectome-level analyses highlighted the importance of preserved global network architecture for recovery. Continued efforts are needed to translate diffusion imaging findings into clinical applicable biomarkers to guide personalized aphasia rehabilitation, with greater use of advanced methods.

## Simple Summary

Post-stroke aphasia represents a significant clinical challenge, as damage to the brain’s white matter is just as critical as damage to the language centers themselves. While diffusion tensor imaging (DTI) is more established, this study reviews how advanced diffusion imaging findings can provide a deeper understanding of white matter changes. We specifically assess the utility of these tools in informing diagnosis, prognostic assessment, and treatment-related recovery in post-stroke aphasia.

## 1. Introduction

Language is a fundamental component of human communication and cognition, and aphasia represents one of the most devastating consequences of stroke, leading to profound impairments in quality of life [1]. Approximately 24–38% of stroke survivors experience aphasia in the acute stage and although varying degrees of spontaneous recovery may occur, 10–18% continue to live with chronic aphasia [2,3]. The loss of white matter integrity is implicated in the majority of cerebral infarctions, arising either from direct structural damage or from secondary degenerative processes such as Wallerian degeneration, in which white matter integrity deteriorates in regions remote from the primary lesion [4,5].

**Figure 1:**
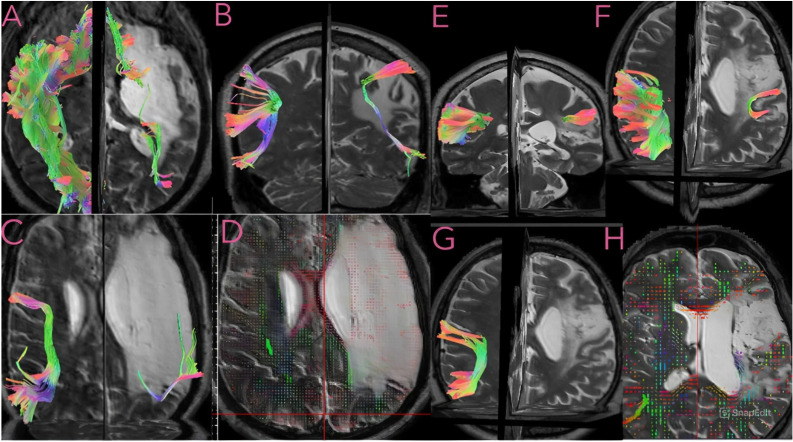
Tractography and quantitative anisotropy (QA) maps of white matter pathways in two patients with chronic stroke. Images (**A**–**D**) correspond to the first patient (52-year-old male, chronic stroke), and images (**E**–**H**) correspond to the second patient (55-year-old male, chronic stroke). In the first patient, (**A**) shows the uncinate fasciculus (UF) and inferior longitudinal fasciculus (ILF); (**B**,**C**) demonstrate the arcuate fasciculus (AF) from different views, with reduced fiber representation compared with the contralateral hemisphere; (**D**) highlights microstructural alterations in perilesional white matter. In the second patient, (**E**,**F**) illustrate the superior longitudinal fasciculus (SLF), which demonstrates reduced fiber representation relative to the contralateral hemisphere; (**G**) shows the arcuate fasciculus (AF), which is not reliably reconstructed; (**H**) highlights microstructural alterations in perilesional white matter. Tracts are represented on a T2-weighted background and categorized by orientation: red (commissural fibers interconnecting homologous regions across hemispheres), green (association fibers linking intra-hemispheric areas), and blue (projection fibers connecting cortical regions with subcortical structures). Data shown in this figure were derived from the Aphasia Recovery Cohort (ARC), an open-source chronic stroke repository [19].

Historically, classical center-based models of localized language function to discrete cortical regions, most prominently Broca’s and Wernicke’s areas. The Broca–Wernicke–Lichtheim–Geschwind model described aphasia as a syndrome resulting from disconnection between Broca’s and Wernicke’s areas [6,7]. These frameworks further identify disconnection syndromes such as transcortical sensory and motor aphasia. Geschwind later proposed that these regions are connected through a single white matter pathway, the arcuate fasciculus [8,9]. Although foundational, these models provided a limited representation of the fiber pathway connectivity supporting language function. Advances in diffusion imaging have enabled in vivo mapping of white matter pathways and characterization of stroke-related microstructural damage, substantially expanding understanding of language network organization. Modern perspectives on language connectivity therefore emphasize multiple interacting association pathways rather than a single tract. The prevailing dual-stream model forms the anatomical foundation of the human language connectome, in which the dorsal and ventral tracts constitute two distinct pathways connecting cortical language regions [10]. Functionally, the dorsal pathway is generally associated with sensorimotor integration and phonological aspects of speech, while the ventral pathway is more closely linked to lexical–semantic processing and language comprehension.The dorsal pathway mainly consists of the arcuate fasciculus (AF) and the superior longitudinal fasciculus (SLF), whereas the ventral pathway is composed of the inferior longitudinal fasciculus (ILF), uncinate fasciculus (UF), inferior fronto-occipital fasciculus (IFOF), and middle longitudinal fasciculus (MdLF) (Figure 1). Diffusion metrics commonly used in post-stroke aphasia research include tensor-derived measures such as fractional anisotropy (FA), reflecting neural tract integrity; radial diffusivity (RD), which increases with demyelination; and mean diffusivity (MD), which tracks reduced membrane density.

Fiber architecture in the human brain is highly complex, with crossing, branching, and kissing fibers often coexisting within a single voxel. For example, the arcuate fasciculus (AF) is characterized by a large proportion of crossing fibers with high posterior curvature, and the inferior fronto-occipital fasciculus (IFOF), inferior longitudinal fasciculus (ILF), and middle longitudinal fasciculus (MdLF) run in close proximity [11,12]. This anatomical complexity highlights the limitations of the diffusion tensor model, which assumes a single dominant fiber orientation per voxel and may therefore lead to inconsistencies and reduced accuracy in clinical–anatomical correlation [13]. To resolve multiple fiber orientations within individual voxels, various methods have been applied in post-stroke aphasia research, including diffusion kurtosis imaging (DKI), constrained spherical deconvolution (CSD) with high-angular-resolution diffusion imaging (HARDI) data, and diffusion spectrum imaging (DSI), each offering distinct microstructural metrics. More advanced tractography algorithms also allow for improved subsegment–function association by more precisely identifying tract segments [14,15,16]. To date, emerging research on global network measures has demonstrated associations with aphasia severity, as well as evidence that preserved global neural network architecture supports neuroplasticity, including right-hemisphere involvement across different stages of recovery. Contemporary network models that unify cortical hubs, white matter connectivity, and large-scale network dynamics offer a principled conceptual link to white matter- and connectome-level diffusion metrics [17,18].

Previous systematic reviews and meta-analyses [20,21,22] have primarily examined language tracts using DTI, providing only a partial view of white matter connectivity. These studies were limited to local structural correlations and could not account for the complex crossing-fiber architecture that underlies language processing. To address this gap, this review synthesizes the clinical applications of established DTI findings while integrating evidence from advanced diffusion imaging methods. Ultimately, this work highlights how advanced techniques contribute to our understanding of white matter integrity and neuroplasticity, demonstrating their superior clinical utility in the context of post-stroke aphasia.

## 2. Materials and Methods

**Figure 2:**
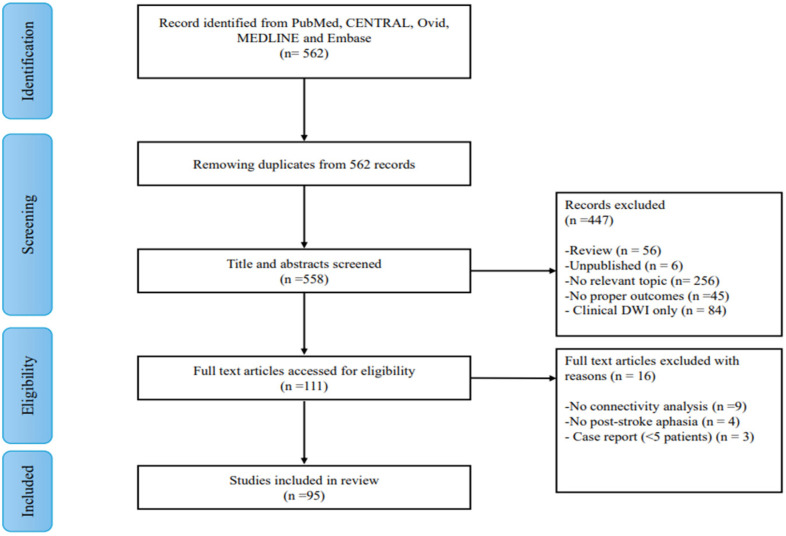
Flowchart of literature retrieval and screening.

The protocol for this review was registered in the Open Science Framework (OSF) to improve transparency and methodological quality (Registration DOI: 10.17605/OSF.IO/6YQFH). A comprehensive search of the English-language literature was conducted in PubMed, CENTRAL, Ovid MEDLINE, and Embase to identify relevant studies from inception to May 2025. We followed PRISMA guidelines, and search strategies were developed using a combination of keywords and standardized index terms: “(aphasia OR language OR language disorders) AND (stroke OR cerebrovascular OR infarct OR blood flow disturbance OR post-stroke) AND (diffusion tensor imaging OR advanced diffusion imaging OR diffusion MRI OR beyond-DTI imaging OR diffusion spectrum imaging OR HARDI OR NODDI OR diffusion tensor MRI)”. The specific search terms are provided in Supplementary File S1. Studies were eligible for inclusion if they reported structural connectivity analyses in PSA, included clinical outcomes in at least one language domain, and involved adults (>18 years of age). Exclusion criteria included conference abstracts, opinion articles, protocols, review articles, systematic reviews, and case reports with fewer than 5 patients. Additionally, we excluded (1) non-human studies; (2) studies without connectivity analysis on diffusion imaging (i.e., those reporting only infarct volume on clinical DWI); (3) duplicate publications; (4) studies on non–stroke-related communication disorders (e.g., dysphagia, dysarthria) or non-language motor impairments; and (5) non-imaging studies. The full search and study selection process is presented in Figure 2.

### 2.1. Study Screening Process

All identified articles were imported into EndNote and Covidence for deduplication and screening. Two independent reviewers (ZK, IY) conducted the initial screening of titles and abstracts to assess eligibility based on the predefined inclusion and exclusion criteria described above. Any disagreements were resolved by consensus between the two reviewers. Articles that passed the initial screening proceeded to full-text review, which was also performed by two independent reviewers (ZK, IY) for final inclusion. Disagreements during this stage were resolved by consensus between two reviewers.

### 2.2. Data Extraction

Data extraction was completed by the two independent reviewers (ZK, IY), and a standardized form was developed for data extraction. Once the data collection form was finalized and formal data extraction was completed, disagreements were resolved by consensus. Extracted variables included participant demographics, stroke and aphasia clinical characteristics, diffusion metrics, tract-specific or connectome measures, language assessment tools and specific language domains, and study design features.

### 2.3. Quality Assessment of Studies

Given the heterogeneity of included study designs, appropriate Joanna Briggs Institute (JBI) critical appraisal tools were applied according to study type, including the Revised Randomized Controlled Trials, Revised Quasi-Experimental Studies, Cohort Studies, Analytical Cross-Sectional Studies, and Case Series checklists. Methodological quality assessments and risk-of-bias judgments categorized by study design are summarized in Supplementary Table S1–S5.

## 3. Results

Following the literature search, 558 studies were identified and included for title and abstract screening. After irrelevant studies were excluded based on this initial screening, 111 studies were retrieved for full-text review. During the full-text screening, 16 studies were excluded due to the absence of diffusion-based connectivity analysis (n = 9), non–stroke-related aphasia (n = 4), or case reports with fewer than five patients (n = 3). A total of 95 [5,14,15,16,17,18,23,24,25,26,27,28,29,30,31,32,33,34,35,36,37,38,39,40,41,42,43,44,45,46,47,48,49,50,51,52,53,54,55,56,57,58,59,60,61,62,63,64,65,66,67,68,69,70,71,72,73,74,75,76,77,78,79,80,81,82,83,84,85,86,87,88,89,90,91,92,93,94,95,96,97,98,99,100,101,102,103,104,105,106,107,108,109,110,111] studies were included for analysis with extractable data. The complete process of searching and including studies is shown in Figure 2. All included studies utilized diffusion MRI to assess structural connectivity and to characterize major language-related white matter pathways, accompanied by correlated language performance measures using various standardized language assessment tools (e.g., WAB, BDAE, BNT, Philadelphia Naming Test). Language function domains were categorized as spontaneous speech, comprehension, repetition, naming, syntax, and reading. Demographic, clinical, and neuroimaging characteristics of the included studies are summarized in Table 1, Table 2 and Table 3.

The included studies were classified into three methodological categories: diagnostic, prognostic, and therapeutic. Cross-sectional investigations examining the relationship between diffusion-derived parameters and language performance were designated as diagnostic. Studies incorporating longitudinal clinical evaluations and/or serial MRI assessments were categorized as prognostic. Investigations implementing active interventions such as speech–language therapy or neuromodulation were assigned to the therapeutic category, with pharmacological studies listed in tables only. Accordingly, the overall findings are organized and synthesized within these three thematic sections. A total of 59 studies were classified under the diagnostic category, 17 addressed prognostic aspects, and 19 were related to therapeutic interventions. The number of enrolled patients with PSA ranged from 5 to 176, with an average of 39.0 participants per study. Modality-specific averages were 41.7 patients for DTI, 26.6 for DSI, 30.6 for CSD, and 22.0 for DKI.

**Table 1:** Demographic, clinical, and neuroimaging characteristics of included diagnostic studies investigating diffusion parameters in post-stroke aphasia.

Study ID	Participant	Imaging	Method	Metric	Anatomical Structure	Language Function
Bonilha 2014 [28]	39 patients	DTI	Connectome analysis	Connectome matrix	Whole-brain connections	Naming
Breier 2008 [30]	20 patients	DTI	Tractography	FA	Right, left AF, ILF, UF	Spontaneous speech, comprehension, repetition, naming
Del Gaizo 2017 [32]	92 patients	DTI	Connectome analysis	Connectome matrix	Language networks	Spontaneous speech, comprehension, repetition, naming
Dickens 2021 [33]	30 patients	CSD	Connectome analysis	Connectome matrix	Whole-brain connections	Reading
Ding 2024 [34]	50 patients	DTI	Connectome analysis	Connectome matrix	Whole-brain connections	Naming, comprehension
Dresang 2021 [35]	14 patients, 15 controls	DSI	Connectome analysis-correlational tractography	QA	AF, MdLF, IFOF, UF, FAT, SLF, CST, and other projection tracts	Naming
Elmongui 2022 [36]	27 patients, 27 controls	DTI	Tractography	FA, MD, fiber count	AF, SLF	Spontaneous speech, comprehension, repetition, naming
Fan 2021 [37]	42 patients, 30 controls	DTI	TBBS	FA	Right hemisphere	Spontaneous speech, comprehension, repetition
Forkel 2018 [38]	159 patients	DTI	Connectome analysis	Connectome matrix	Right-hemisphere connections	Spontaneous speech, comprehension, repetition, naming, syntax, reading
Geva 2015 [41]	15 patients, 18 controls	DTI	Tractography, TBBS	FA	Whole-brain connections, left and right AF	Comprehension, repetition, naming
Gleichgerrcht 2015 [42]	38 patients	DTI	Connectome analysis	Connectome matrix	Whole-brain connections	Spontaneous speech, syntax, naming
Gleichgerrcht 2016 [43]	44 patients	DTI	Connectome analysis	Connectome matrix	Whole-brain connectomes	Comprehension, repetition, naming
Griffiths 2013 [44]	16 patients, 14 controls	DTI	Tractography	FA, MD, RD, AD	AF, EmC	Syntax
Guo 2007 [45]	5 patients	DTI	Tractography	FA	AF	Repetition
Han 2024 [46]	28 patients, 15 controls	DSI	Differential tractography, connectome analysis	QA	Whole-brain connectomes	Spontaneous speech, naming, repetition, comprehension, reading
Han 2016 [47]	69 patients	DTI	Atlas-based	FA-ROI analysis	Major white matter tracts	Naming, comprehension
Harvey 2015 [48]	18 patients	DTI	Atlas-based	FA, ROI-based	IFOF, ILF, UF	Naming, comprehension
Harvey 2013 [49]	10 patients, 8 controls	DTI	Tractography	Delineation	IFOF, ILF, UF	Naming, comprehension
Hosomi 2009 [50]	13 patients	DTI	Tractography	FA	AF	Spontaneous speech, repetition
Hula 2020 [51]	42 patients	DSI	Connectome analysis	QA	Whole-brain connectomes	Naming
Ivanova 2016 [52]	37 patients, 11 controls	DTI	Atlas-based	FA, MD, AD, RD	AF, SLF, ILF, IFOF, UF, CC, CST	Comprehension, syntax, naming
Ivanova 2021 [15]	33 patients	CSD-HARDI	Tractography	HMAO, normalized volume	AF	Spontaneous speech, comprehension, repetition, naming
Keator 2021 [54]	97 patients	DTI	Connectome analysis	Fiber count	Whole-brain connectomes	Spontaneous speech, comprehension, repetition, naming
Keser 2023 [55]	56 patients, 10 controls	DTI	Tractography	FA, MD	Cerebellum, corticocerebellar connection	Spontaneous speech, comprehension, repetition, naming
Keser 2021 [5]	61 patients	DTI	Tractography	MD	DMVAC, PLC, thalamocortical projections	Naming
Kim 2011 [59]	12 patients	DTI	Tractography	Delineation, FA	AF	Spontaneous speech, comprehension, repetition, naming
Kourtidou 2021 [60]	25 patients, 24 controls	DTI	Tractography	FA, AD, RD	SLF II and SLF III, TFexcF, AF	Spontaneous speech, comprehension, repetition, naming
Koyama 2016 [61]	10 patients, 21 controls	DTI	TBBS, tractography	FA	AF	Spontaneous speech, comprehension, repetition, naming
Kristinsson 2021 [62]	116 patients	DTI	Atlas-based ROI	FA	Left hemisphere	Spontaneous speech, comprehension, repetition, naming
Kyeong 2019 [63]	40 patients	DTI	Whole-brain regression	FA	Whole brain	Spontaneous speech, comprehension, repetition, naming
Lee 2021 [64]	64 patients	DTI	Tractography	FA	AF, ILF, UF, IFOF, SLF	Spontaneous speech, comprehension, repetition, naming
Lee 2018 [66]	23 patients, 10 controls	DTI	VBM	FA	Whole brain	Spontaneous speech, comprehension, repetition, naming
Martinez Oeckel 2021 [69]	123 patients	DTI	Tractography	Tract lesion load	AF, EmC	Comprehension
Matchin 2024 [70]	103 patients	DTI	Connectome analysis	Connectome matrix	Left-hemisphere connectomes	Syntax
McCall 2022 [71]	51 patients, 37 controls	CSD	Connectome analysis	Connectome matrix	Whole-brain connectomes	Spontaneous speech, naming, comprehension
McKinnon 2018 [72]	32 patients	DKI	Tractography	AWF	Dorsoventral stream tracts	Naming
Medaglia 2022 [74]	39 patients, 36 controls	DTI	Connectome analysis	Connectome matrix	Whole-brain connectomes	Spontaneous speech, comprehension, repetition, naming
Noh 2021 [77]	41 patients	DTI	Tractography	FA, AD, MD, RD, tract volume	AF	Spontaneous speech, comprehension, repetition, naming
Olive 2023 [78]	19 patients	DTI	Tractography	Tract volume, FA	AF, ILF, UF and IFOF	Repetition
Olson 2025 [79]	14 patients, 15 controls	DSI	Tractography (differential and correlational), connectometry	QA	AF, FAT, IFOF, ILF, SLF, EmC, Cingulum	Naming
Papoutsi 2011 [81]	14 patients, 15 controls	DTI	ROI-based tractography	FA, MD	AF, EmC	Syntax
Rolheiser 2011 [82]	24 patients, 9 controls	DTI	Atlas-based, ROI tractography	FA	AF, EmC, whole brain	Comprehension, syntax, repetition
Rosso 2015 [84]	23 patients	DTI	Whole-brain regression	FA	Whole brain	Spontaneous speech, comprehension, repetition, naming, syntax
Roth 2024 [86]	87 patients	DTI	Connectome analysis	Fiber length	Whole brain	Spontaneous speech, comprehension, repetition, naming
Salvalaggio 2020 [87]	176 patients	DTI	Atlas-based connectome analysis	Connectome	Whole-brain connections	Spontaneous speech, comprehension, reading
Soliman 2023 [94]	17 patients, 10 controls	CSD	Tractography	FA, MD, AD and RD	AF, IFOF, ILF, UF, FAT	Comprehension, repetition, naming
Tak 2014 [96]	25 patients	DTI	Tractography	FA, ADC, volume	AF	Spontaneous speech, Naming, Comprehension, Repetition
Wang 2020 [98]	15 patients, 9 controls	DTI	Tractography	FA	AF	Repetition
Xiao 2024 [100]	79 patients, 41 controls	DTI	Connectome analysis	Connectome matrix, nodal degree	Whole-brain connections	Naming, comprehension
Xing 2017 [101]	40 patients, 27 controls	DTI	TBBS	FA, AD, MD, RD	Whole-brain connections	Comprehension
Xing 2018 [102]	45 patients	DTI	Connectome analysis	FA	Whole-brain connections	Naming
Yang 2017 [103]	18 patients, 20 controls	DTI	TBBS	FA	Whole-brain connections	Spontaneous speech, comprehension, repetition, naming
Yourganov 2016 [104]	90 patients	DTI	Connectome analysis	Fiber count	Whole-brain connections	Spontaneous speech, comprehension, repetition, naming
Yu 2023 [105]	38 patients	DTI	Tractography	FA	AF, UF	Spontaneous speech, comprehension, repetition, naming
Yu 2022 [106]	26 patients	DTI	Tractography	FA, fiber count	AF	Spontaneous speech, comprehension, repetition, naming
Zhang 2018 [108]	14 patients, 11 controls	DTI	TBBS, tractography	FA, AD, MD, RD	AF, SLF, UF, IFOF, ILF	Spontaneous speech, comprehension, repetition, naming, reading
Zhang 2021a [14]	29 patients, 33 controls	CSD	Tractography	FD, bundle cross section	SLF III, AF, IFOF, UF, ILF, MdLF, FAT	Spontaneous speech, comprehension, repetition, naming, reading
Zhoung 2022 [109]	33 patients	CSD-HARDI	Tractography	HMAO, normalized volume	FAT	Spontaneous speech, comprehension, repetition, naming, motor speech
Zyryanov 2020 [110]	20 patients	DTI	Tractography	FA, MD, RD, volume	FAT	Naming, syntax

**Table 2:** Demographic, clinical, and neuroimaging characteristics of included prognostic studies investigating diffusion parameters in post-stroke aphasia.

Study ID	Participant	Imaging	Method	Metric	Anatomical Structure	Language Function	Assessment Point
Agrawal 2024 [23]	36 patients	DTI	Tractography	LI-FA, LI-MD	Right, left AF	Spontaneous speech, comprehension, repetition, naming	One scan, two language assessments
Rong Bae 2022 [25]	35 patients	DTI	Tractography	FA, MD, RD, AD	Right, left AF	Spontaneous speech, comprehension, repetition, naming	Two scans, two language assessments
Blom-Smink 2020 [26]	10 patients	DTI	Tractography	FA	Right SLF, IFOF, ILF, MdLF, UF	Naming	Two scans, two language assessments
Forkel 2018 [38]	18 patients	DTI	Tractography, VLSM, TBBS	AP, FA	Right, left AF, FAT, IFOF, UF, whole-brain connections	Spontaneous speech, comprehension, repetition, naming	One scan, two language assessments
Forkel 2014 [39]	18 patients	DTI	Tractography	Tract volume	AF	Spontaneous speech, comprehension, repetition, naming	One scan, two language assessments
Jang2017 [53]	18 patients	DTI	Tractography	FA, delineation	AF	Spontaneous speech, comprehension, repetition, naming	Two scans, two language assessments
Keser2020a [56]	28 patients	DTI	Tractography	FA, RD	AF, IFOF, ILF	Spontaneous speech, comprehension, syntax, comprehension	Three scans, three language assessments
Keser2020b [57]	24 patients	DTI	Tractography	FA, MD	Right AF, FAT	Naming	Two scans, two language assessments
Kim2013 [58]	25 patients, 12 controls	DTI	Tractography	Delineation	AF	Spontaneous speech, comprehension, repetition, naming	One scans, two language assessments
Lee2020 [65]	68 patients	DTI	Tractography	LI, AF, MD, AD, RD	AF	Spontaneous speech, comprehension, repetition, naming	Two scans, two language assessments
Leo 2019 [67]	14 patients, 17 controls	DTI	VBM, tractography	FA	Whole brain	Spontaneous speech, comprehension, repetition, naming	One scan, two language assessments
Moulton 2019 [76]	28 patients	DTI	ROI-based tractography	AD-ROI-based	AF, IFOF, ILF, UF	Spontaneous speech, comprehension, repetition, naming	One scan, two language assessments
Osa Garcia 2024 [80]	39 patients	DTI	Tractography	FA, MD, AD	IFOF, UF, and ILF, AF	Comprehension, repetition, Naming	One scan and three language assessments
Schevenels 2022 [16]	31 patients	CSD-HARDI	Tractography	FBC	AF, IFOF	Repetition, naming, comprehension	Two scans, three language assessments
Sihvonen 2022a [90]	39 patients	DTI	Tractography	FA	AF, IFOF, CC, tapetum	Spontaneous speech	Two scan and two language assessments
Sihvonen 2023 [92]	22 patients	DSI	Connectome analysis	QA	Right-hemisphere connections	Spontaneous speech, naming, comprehension	One scan, two language assessments
Zhang 2021b [111]	36 patients, 24 controls	DTI	Tractography	FA	UF	Spontaneous speech, comprehension, repetition, naming	Two scans, two language assessments

**Table 3:** Demographic, clinical, and neuroimaging characteristics of included therapeutic studies investigating diffusion parameters in post-stroke aphasia.

Study ID	Participant	Imaging	Method	Metric	Anatomical Structure	Language Function	Treatment
Allendorfer 2012 [24]	8 patients	DTI	TBBS	FA	Left hemisphere	Spontaneous speech, comprehension, naming	rTMS
Bonilha 2016 [27]	24 patients	DTI	Connectome analysis	Connectome matrix	Whole-brain connections	Naming	Speech–language therapy
Braun 2022 [29]	34 patients	DTI	Tractography	FA, MD	Right, and left AF, SLF, ILF, IFOF, UF	Spontaneous speech, comprehension, repetition, naming	Speech–language therapy
Chang 2021 [31]	26 patients	DKI	Atlas-based	Mean Kurtosis	Gray matter structures	Naming	tDCS
Lin 2023 [68]	33 patients	DTI	Tractography	FA, AD, RD, ADC	AF	Spontaneous speech, comprehension, repetition, naming, writing	LF-rTMS
Low 2024 [18]	16 patients	DTI	Connectome analysis	Modularity, participation coefficients	Language and general networks	Naming	Speech–language therapy
McKinon 2017 [73]	8 patients	DKI	Tractography	MK	Dorsoventral stream tracts	Naming	Speech–language therapy
Meier 2019 [75]	34 patients	DTI	Atlas-based	ROI-based	Whole-brain gray and white matter	Spontaneous speech, comprehension, repetition, naming, syntax	Speech–language therapy
Rosso 2014 [83]	25 patients	DTI	Tractography	FA	AF, FAT, IFOF	Naming	tDCS
Roth 2023 [85]	78 patients	DTI	Connectome analysis	Structural network integrity	Whole brain	Naming	Speech–language therapy
Sihvonen 2021 [86]	38 patients	DTI	Tractography	FA	FAT	Spontaneous speech, naming, Comprehension	Music therapy
Sihvonen 2024 [88]	28 patients	DSI	Connectome analysis	QA	Whole-brain connections	Naming	Singing therapy
Sihvonen 2022b [91]	38 patients	DSI	Connectome analysis	QA	Whole-brain connections	Spontaneous speech, Naming, Comprehension	Music therapy
Soliman 2021 [93]	21 patients	CSD	Tractography	FA, MD	AF, IFOF, ILF, UF, FAT	Comprehension, repetition, naming	tDCS
Stockbridge 2024 [95]	58 patients	DTI	Atlas-based	MD	AF, IFOF, ILF, SLF	Spontaneous speech, naming, comprehension	Speech–language therapy
Wan 2014 [97]	11 patients	DTI	Individual ROI	FA, AD, RD	Right hemisphere	Spontaneous speech	Speech–language therapy
Wang 2025 [99]	16 patients	DTI	TBBS	FA	Whole-brain connections	Spontaneous speech, comprehension, repetition, naming	Venlafaxine
Wilmskoetter 2022 [17]	68 patients	DTI	Connectome analysis	Global controllability, regional contractibility	Whole-brain connections	Naming	Speech therapy+AtDCS
Yu 2019 [107]	20 patients	DTI	Tractography	FA, fiber count	AF	Spontaneous speech, comprehension, repetition, naming	Speech–language therapy

Among the 95 included studies, diffusion MRI methodology varied substantially. Seventy-seven studies employed conventional DTI, whereas 18 used advanced models (CSD n = 8; DSI n = 7; DKI n = 3). With respect to analytical approach, tractography was most common (n = 55), followed by connectome-based methods (n = 27) and other or mixed methods, including voxel-based, ROI-based, or atlas-based segmentation analyses (n = 13). Forty-five studies focused exclusively on at least one dorsal or ventral language pathway, whereas fifty-one studies examined whole-brain connectivity, interhemispheric connections, or additional non-language-specific tracts. The literature reflects a substantial range of analytical frameworks, including approaches defined by both methodological design and underlying neuroanatomy. These perspectives are critical for constructing an integrated model of language production and recovery, but they also elucidate the divergence in findings observed across different analytical strategies and studies. Tract-specific diagnostic, prognostic, and therapeutic associations across studies are summarized in Table 4.

## 4. Discussion

This review integrates diffusion imaging findings to delineate how white matter disconnection and network reorganization shape language impairment, recovery, and treatment response in post-stroke aphasia.

### 4.1. Diagnostic Utility of Diffusion Imaging

#### 4.1.1. Relationship Between Aphasia Severity, Impairment Types, and Diffusion Imaging Biomarkers

Reduced microstructural integrity of left-hemisphere tracts—compared to right-hemisphere homologues and to left-hemisphere tracts in controls—has been used to investigate the relationship between diffusion metrics and language deficits, serving a diagnostic purpose by identifying structural biomarkers of language network damage underlying PSA [51,102]. Clinical measures of motor–speech impairment are most often associated with dorsal-stream damage, whereas impaired speech comprehension more strongly involves the ventral stream. Behaviors such as naming, speech repetition, and grammatical processing rely on interactions between the two streams [40,44,82]. Consistent with this interaction-based organization, evidence from DKI, CSD, and DSI supports network-level contributions across dorsal and ventral pathways to naming performance [35,51,72]. Similarly, syntactic processing has been linked to distributed language network integrity, with growing evidence highlighting contributions from verbal working memory systems [70,81,82].

Cross-sectional tractography and connectome analyses have demonstrated associations between structural and diffusion metrics and overall aphasia severity, typically assessed by the WAB Aphasia Quotient (AQ). Diagnostic markers differ based on stroke stage. In the early stage, structural markers are broader, including LI–MD of the AF [23], left AF FA changes [105], and right FAT volume [38]. In the subacute stage, associations have been reported for LI–FA of the AF [23]. In the chronic stage, studies have highlighted the role of left AF delineation [53,59] and left AF FA changes [61]. Although results vary across studies, the integrity and preservation of left AF lateralization remain consistently important for overall language function across all stroke stages.

While the left AF serves as a robust local marker, expanding the focus to global networks provides critical insight, particularly in the chronic stage when network organization is more stable. Connectome analyses have shown that temporoparietal-junction connectivity [32], spared white matter connections [104], a higher proportion of long-range fibers [85], and increased nodal participation [42] are each associated with reduced aphasia severity. These findings emphasize that global network preservation contributes to language outcomes independently of local tract damage.

#### 4.1.2. Tract-Specific Abnormalities: Conventional Approach and Emerging Targets

Subsegment analysis from advanced diffusion methods underscores the multifaceted role of the AF, reflecting its inherent fiber complexity and extending beyond repetition and syntactic production. Specific components of the AF contribute differentially to language processing [107]. The anterior segment has been associated with conceptually driven speech production and fluency [41,98], whereas the posterior segment is crucial for lexical–semantic processing and comprehension at the word and sentence levels [15,41,52,94,106].

In contrast to the AF, the frontal aslant tract (FAT) is a relatively new focus, and its function is still being investigated. A CSD-based study normalized tract volume reported an association between the FAT and motor-speech function, consistent with earlier work identifying its role in speech initiation and verbal fluency [109]. In contrast, the FAT has not been found to be associated with lower-level conceptual or lexical selection processes [110].

While these findings use advanced methods to refine the roles of dorsal pathways (AF and FAT), a complete structural framework requires capturing the systemic impact of stroke that extends beyond the primary lesion. Damage can spread to remote regions through disruption of long-range white matter pathways, as demonstrated in a large cohort of 97 patients [54]. Wallerian degeneration has been proposed as a mechanism leading to anterograde and retrograde reductions in microstructural integrity [55], linking focal injury to abnormalities in distant regions and demonstrating how secondary degeneration shapes network organization in PSA. Recent aphasia studies using DTI have also identified tracts affected by secondary degeneration outside the dorsal–ventral language pathways that contribute to language outcomes. Keser et al. [55] examined a large group (n = 56), similar in scale to earlier work, and reported reduced microstructural integrity with lower FA and higher MD values in the right cerebellum and its connections to left cortical regions. Similarly, loss of thalamocortical pathway integrity has been correlated with poorer picture naming in the late-stroke group. Secondary degeneration, which becomes more pronounced in the chronic stage, may further influence aphasia outcomes [5].

Differential tractography studies using group-level comparisons between patients and healthy controls have demonstrated that verb retrieval accuracy is associated with differences in QA across projections and commissural pathways. Multiple cortico-subcortical projection pathways [35,79], the corpus callosum [35,46] and limbic pathways supporting semantic and phonological processes during word production have been implicated [51]. Damage to limbic pathways also contributes to post-stroke basal ganglia aphasia, which presents impaired word fluency and naming, as well as emotional and cognitive dysfunction [46]. These findings emphasize the need for future diagnostic frameworks to incorporate higher-order neural networks that support language functions as part of the structural basis of PSA.

### 4.2. Prognostic Utility

#### 4.2.1. Quantitative Metrics as Prognostic Markers in Various Stages After Stroke

Diffusion imaging biomarkers have shown promise in predicting long-term language outcomes. However, questions remain regarding which specific markers have the greatest predictive utility and how these markers operate independently of total lesion volume. These biomarkers may also vary across stages of PSA, as stage-dependent network reorganization influences diffusion metrics. Previous studies have used both early diffusion imaging to predict long-term language outcomes and repeated imaging in longitudinal designs.

Early-stage DTI studies have emphasized the prognostic role of left AF integrity [50,53,56,58], with some extending this line of evidence to right AF integrity at six months [38,39] and the predictive value of left AF integrity at three months [76]. More recently, Osa García et al. [80], in a cohort of 39 patients with aphasia, demonstrated differential contributions of white matter tracts over time: early left IFOF integrity (within 10 ± 3 days post-stroke) predicted early improvement, whereas later AF integrity was more predictive of six-month language outcomes. CSD-based evidence found no specific contribution of acute dual-stream connectivity to the prediction of later language outcomes [16]. Such discrepancies may arise from methodological heterogeneity across studies, particularly in the timing of imaging and clinical assessments. Optimal time points for capturing stable predictive markers and the accuracy of long-term outcome estimates based on early scans remain uncertain.

Importantly, FA changes in the acute stage of ischemia are strongly influenced by cytotoxic edema, which reduces diffusivity and limits the reliability of early DTI metrics [112]. Similarly, CSD-based analyses may yield negative or unstable results in the acute phase because free water from edema introduces noise and reduces the reliability of fiber-orientation estimates [113].

Taken together, although several tract-level diffusion metrics have demonstrated prognostic relevance, their results are highly dependent on scan timing, analytical approach, and the pathophysiological state of the tissue.

#### 4.2.2. Longitudinal Tracking with Diffusion Imaging

While early-stage imaging provides insight into potential outcomes, longitudinal studies are essential for capturing the dynamic evolution of white matter changes during recovery. Emerging diffusion-imaging studies suggest that the integrity and connectivity of spared neural tissue play a central role in aphasia recovery. The degree to which brain networks are preserved and/or undergo microstructural reorganization beyond the lesion appears to be a key factor influencing aphasia severity and recovery potential [86].

A longitudinal DTI study of 39 patients spanning 1 to 6 months post-stroke showed a decrease in bilateral AF integrity, but only changes in left AF (FA and RD) were associated with poorer language outcomes [25]. Blom-Smink et al. [26], in a smaller cohort of 10 patients, observed that increases in FA within the right ILF over a one-month interval were associated with significant improvements in naming accuracy. These findings suggest that bilateral tracts undergo experience-dependent microstructural change during the recovery period and that such changes may correlate with improvements in language performance.

#### 4.2.3. Right-Hemisphere Contributions to Language Recovery

Left-hemisphere integrity and global network reorganization are central to post-stroke recovery, but the compensatory role of the right hemisphere remains controversial. Some evidence suggests that right-hemisphere tracts may support residual language processing. For example, two acute-phase studies [39,80] showed that right AF integrity predicted less severe chronic aphasia. Similarly, preserved or compensatory structural integrity in right-hemisphere tracts has been linked to improved language outcomes in some cases [60,66]. However, other studies indicate that recruitment of the right hemisphere may be maladaptive. Keser et al. [57] demonstrated that transfer of language function to the right hemisphere was associated with poorer language outcomes.

Diffusion spectrum imaging (DSI) may help explain these inconsistencies by revealing differences in lateralization across language pathways. Ventral pathways such as the IFOF tend to be more bilaterally organized, whereas dorsal pathways such as the AF are more strongly left-lateralized [114,115,116]. Connectome-level analyses by Sihvonen et al. [92] showed that increased right-hemisphere connectivity in the early subacute stage was negatively associated with language production but positively associated with comprehension, suggesting domain-specific effects. Positive associations reported for the posterior corpus callosum and right IFOF involvement in semantic processing further support a role for right-hemisphere contributions during the recovery period [51].

Individual variability plays a key role in determining the functional relevance of right-hemisphere engagement. Factors such as stroke severity, lesion location, and time post-onset may influence whether right-sided recruitment supports or hinders recovery [66].

### 4.3. Treatment Planning and Monitoring

#### 4.3.1. Imaging Biomarkers of Treatment Response

Treatment outcomes in post-stroke aphasia vary considerably, with some patients demonstrating substantial improvement while others exhibit only limited or minimal progress. Identifying which patients are most likely to benefit from therapy remains a critical challenge. Pre-treatment diffusion imaging markers may offer valuable information for guiding individualized therapies, and post-treatment changes can provide insight into underlying neuroplastic mechanisms and inform future interventions.

Several longitudinal studies suggest that white matter integrity prior to therapy is associated with treatment response. Greater preservation of global networks [28], higher network modularity [18], and intact ventral tracts [75] have all been associated with more favorable responses to various speech–language therapy interventions, underscoring the critical role of intact white matter pathways in supporting recovery. Additionally, longitudinal QA changes in the AF and FAT following music therapy suggest tract-specific remodeling [88]. Similarly, increased FA in the left ILF [29] and increased FA in the left FAT [89] support the idea of widespread microstructural reorganization underlying therapy-induced recovery.

#### 4.3.2. Use of Diffusion Imaging in Neuromodulation Studies

Non-invasive brain stimulation techniques have been increasingly used to enhance language recovery in post-stroke aphasia. Previous evidence showed that the stimulation effect was not restricted to the targeted area but modulated a larger network to support language improvement in PSA. Structural remodeling of these networks following stimulation may correlate with improvements in language performance.

Recent studies have demonstrated that pre-treatment white matter integrity can predict responsiveness to neuromodulation. For example, a randomized, double-blind, sham-controlled trial including 33 patients with chronic post-stroke aphasia demonstrated that higher pre-treatment integrity of the right arcuate fasciculus was associated with a greater language response to low-frequency rTMS [68]. Similarly, a randomized sham-controlled crossover tDCS study showed that naming improvement following right-hemisphere stimulation depended on preserved arcuate fasciculus integrity in chronic post-stroke aphasia [83]. In another randomized sham-controlled study involving 22 patients with DTI data collected within the first three months post-stroke, baseline microstructural integrity of the left posterior middle temporal gyrus, arcuate fasciculus, and superior longitudinal fasciculus predicted naming recovery in response to anodal tDCS combined with speech therapy [95]. Extending these findings, a randomized sham-controlled trial of 21 patients reported that increased FA in the right UF was associated with improved fluency following bi-hemispheric tDCS, with significant changes observed only in the real stimulation group compared with sham [93].

Network-level analyses have provided deeper insights into stimulation-induced recovery. Specifically, a connectome analysis using network control theory found that the controllability of the inferior frontal regional network predicted recovery in language production in participants receiving standardized language therapy with adjunctive A-tDCS or sham stimulation, independent of stimulation assignment [17], underscoring the importance of network connectivity.

### 4.4. Other Challenges

One of the limitations in diffusion imaging is the interplay between lesion load and location (stroke-related infarct and white matter hyperintensities) and structural connectivity. Lesion load in critical language-relevant regions is strongly associated with both language outcomes and structural connectivity measures. Determining whether outcomes result from structural disconnection rather than lesion load is difficult because of the confounding effect. Although diffusion studies applied lesion masking to exclude damaged voxels and statistically controlled for total lesion volume, controlling for lesion size remains complex [117].

In this review, we included studies spanning all network scales (whole brain and language network levels), metric types (tract-based and network-based), and methodological approaches (direct versus indirect measures). Connectome analysis is grounded in mathematical modeling, whereas tractography relies on neuroanatomical principles.

A limitation is the substantial heterogeneity in diffusion MRI acquisition and processing protocols. Studies employed widely varying b-values, numbers of diffusion-encoding directions, single- or multi-shell acquisition schemes, MRI field strengths, and preprocessing pipelines, in addition to different tractography algorithms and software platforms. This methodological variability leads to differences in derived microstructural measures across studies and undermines the reliability of pooled or generalized conclusions.

Despite the growing use of conventional DTI and advanced diffusion techniques, their applicability is limited by extensive acquisition requirements and increased data demands. These constraints result in longer overall scan times compared with conventional DTI and preclude routine implementation in research and clinical protocols, especially for advanced diffusion techniques. As reflected in the included literature, studies using advanced diffusion methods tend to enroll smaller cohorts than DTI studies, which may increase susceptibility to publication bias and thereby restrict the reproducibility and generalizability of findings

## 5. Conclusions

In this review, we comprehensively examined diffusion imaging studies focusing on the language connectome in stroke patients, specifically assessing their potential utility for informing diagnosis, prognosis assessment, and treatment-related recovery of aphasia. The existing literature reveals the interdependent and specialized roles of dorsoventral tracts in different domains of language. Most studies used conventional DTI, but emerging evidence suggests a superior utility and improved fiber-specific sensitivity of advanced diffusion techniques in language network research. Critically, current evidence shows that successful language recovery relies not solely on the local microstructural integrity of the dorsal and ventral tracts but also on the preservation of global network architecture and remote cortico-subcortical connectivity. Despite the extensive focus on left-hemisphere association pathways, the contributions of commissural and projection systems remain relatively underexplored and represent important targets for future studies. However, clinical translation of diffusion imaging findings remains limited.

## Figures and Tables

**Table 4:** Left-hemisphere tracts within the language network and summary of tract-specific language outcomes in post-stroke aphasia.

Language Tract	Diagnostic	Prognostic	Therapeutic
**AF**	Associated with aphasia severity and multiple language domains	Left AF integrity and laterality indices predict language outcomes across stroke stages	Microstructural changes observed following language therapy and neuromodulation
**SLF**	Related to phonological processing and repetition deficits	Limited longitudinal prognostic evidence	Included in studies predicting response to language therapy and neuromodulation
**ILF**	Implicated in semantic processing and naming impairment	Limited longitudinal prognostic evidence	Microstructural changes reported following language therapy
**IFOF**	Associated with comprehension deficits and naming impairment	Limited prognostic associations with lexical–semantic outcomes	Included in studies predicting response to language therapy and neuromodulation
**UF**	Related to lexical–semantic deficits and naming impairment	Limited longitudinal prognostic evidence	Microstructural changes observed following language therapy and neuromodulation

## Data Availability

The data used in this study are publicly available from the Aphasia Recovery Cohort (ARC), an open-source chronic stroke repository hosted on OpenNeuro (https://openneuro.org/datasets/ds004884/versions/1.0.1), accessed on 1 February 2026.

## Supplementary Materials

The following supporting information can be downloaded at: https://www.mdpi.com/article/10.3390/tomography12020028/s1, Supplementary File S1: Database search strategies and terms; Supplementary File S2: The PRISMA 2020 Checklist [118]; Supplementary Tables S1–S5: Joanna Briggs Institute critical appraisal checklists for randomized controlled trials, quasi-experimental studies, analytical cross-sectional studies, analytical cohort studies, and case series.

## Abbreviations

**AD**	Axial Diffusivity
**ADC**	Apparent Diffusion Coefficient
**AF**	Arcuate Fasciculus
**AQ**	Aphasia Quotient
**AWF**	Axonal Water Fraction
**BDAE**	Boston Diagnostic Aphasia Examination
**BNT**	Boston Naming Test
**CC**	Corpus Callosum
**CSD**	Constrained Spherical Deconvolution
**CST**	Corticospinal Tract
**DKI**	Diffusion Kurtosis Imaging
**DSI**	Diffusion Spectrum Imaging
**DTI**	Diffusion Tensor Imaging
**EC**	Extreme Capsule
**EmC**	Extreme Capsule
**FA**	Fractional Anisotropy
**FAT**	Frontal Aslant Tract
**FBC**	Fiber Bundle Capacity
**FD**	Fiber Density
**HARDI**	High-Angular-Resolution Diffusion Imaging
**HMOA**	Hindrance-Modulated Orientation Anisotropy
**IFOF**	Inferior Fronto-Occipital Fasciculus
**ILF**	Inferior Longitudinal Fasciculus
**LI**	Laterality Index
**MD**	Mean Diffusivity
**MdLF**	Middle Longitudinal Fasciculus
**MK**	Mean Kurtosis
**NODDI**	Neurite Orientation Dispersion and Density Imaging
**OSF**	Open Science Framework
**PRISMA**	Preferred Reporting Items for Systematic Reviews and Meta-Analyses
**PSA**	Post-Stroke Aphasia
**QA**	Quantitative Anisotropy
**RD**	Radial Diffusivity
**ROI**	Region of Interest
**rTMS**	Repetitive Transcranial Magnetic Stimulation
**SLF**	Superior Longitudinal Fasciculus
**tDCS**	Transcranial Direct Current Stimulation
**TBSS**	Tract-Based Spatial Statistics
**UF**	Uncinate Fasciculus
**VLSM**	Voxel-Based Lesion-Symptom Mapping
**WAB**	Western Aphasia Battery
