# Supplementary material for: Diffusion Tensor Imaging and Advanced Diffusion Imaging in Post-Stroke Aphasia Recovery
Source: Tomography. 2026 Feb 23;12(2):28. doi: 10.3390/tomography12020028 (PMC12944503; doi:10.3390/tomography12020028)
Supplement: Supplementary file 1 [file tomography-12-00028-s001.zip › Supplementary File S1.pdf]

Ovid

Database(s): APA PsycInfo 1806 to May 2025 Week 1, EBM Reviews - Cochrane Central Register of Controlled Trials April 2025, EBM Reviews - Cochrane Database of Systematic Reviews 2005 to May 7, 2025, Embase 1974 to 2025 May 09, Ovid MEDLINE(R) and Epub Ahead of Print, In-Process, In-Data-Review & Other Non-Indexed Citations, Daily and Versions 1946 to May 09, 2025
Search Strategy:

#	Searches	Results
1	exp Aphasia/	74116
2	exp Language Disorders/ or Language/	394460
3	(Acalculia or agnosia* or Agrammatism or Agraphia* or alogia* or anepia* or anomia* or anomy or anosognosia* or aphasia or aphasia* or "dejerine lichtheim phenomenon" or dysphasia* or "Landau Kleffner" or language or "language impairment*" or "language outcome*" or "language recovery" or "lichtheim sign" or "lichtheims sign" or linguistic or logagnosia* or logamnesia* or logasthenia* or "mesulam syndrome*" or "mesulams syndrome*" or naming or "post-stroke aphasia" or prosopagnosia* or "semantic dementia*" or "temporal variant frontotemporal dementia*" or "temporal variant FTD" or "temporal variant of frontotemporal dementia*" or "temporal variant of FTD" or tvFTD or "Word Deaf*").ti,ab,kf.	969148
4	1 or 2 or 3	1110074
5	exp Stroke/	729292
6	(((cerebral or brain or cerebrovascular* or "cerebral vascular*" or "cerebrum vascular*") adj3 (insult or insultus or accident* or "blood flow disturbance*" or infarct* or ischem* or ischaem*)) or ((cerebrovascular* or "cerebral vascular*" or "cerebrum vascular*") adj3 (event* or disorder*)) or apoplexia or apoplexy or "ischaemic seizure*" or "ischemic seizure*" or poststroke* or "post-stroke*" or stroke or strokes).ti,ab,kf.	1242502
7	5 or 6	1383410
8	4 and 7	47740
9	exp Diffusion Tensor Imaging/	56802
10	("advanced diffusion imaging" or "beyond-DTI imaging" or "diffusion kurtosis imaging" or "diffusion MRI" or "diffusion MRIs" or "diffusion tensor imaging" or "diffusion tensor magnetic resonance imaging" or "diffusion tensor mri" or "diffusion tensor mris" or "diffusion tractograph*" or "diffusional spectral imaging" or "diffusion-weighted imaging" or "diffusion-weighted magnetic resonance imaging" or "diffusion-weighted MRI" or "diffusion-weighted MRIs" or "dti mri" or "dti mris" or HARDI or NODDI or tractograph* or "white matter tractography").ti,ab,kf.	128698
11	9 or 10	139749
12	8 and 11	1174
13	limit 12 to english language [Limit not valid in CDSR; records were retained]	1130

14	limit 12 to no language specified [Limit not valid in APA PsycInfo,CDSR; records were retained]	116
15	13 or 14	1130
16	(newborn* or neonat* or infant* or toddler* or child* or adolescent* or paediatric* or pediatric* or girl or girls or boy or boys or teen or teens or teenager* or preschooler* or "pre-schooler*" or preteen or preteens or "pre-teen" or "pre-teens" or youth or youths).ti,ab,hw,kf.	12102411
17	(adult or adulthood or adults or centenarian* or elderly or geriatric* or "middle age" or "middle aged" or nonagenarian* or octogenarian* or "old adult*" or "old people" or "old person*" or "older adult*" or "older people" or "older person*" or septuagenarian* or Sextenarian* or "very old").ti,ab,hw,kf.	22699002
18	16 not 17	7302144
19	15 not 18	1090
20	(exp animals/ or exp nonhuman/) not (exp humans/ or exp patient/)	12995947
21	((alpaca or alpacas or amphibian or amphibians or animal or animals or antelope or armadillo or armadillos or avian or baboon or baboons or beagle or beagles or bee or bees or bird or birds or bison or bovine or buffalo or buffaloes or buffalos or "c elegans" or "Caenorhabditis elegans" or camel or camels or canine or canines or carp or cats or cattle or chick or chicken or chickens or chicks or chimp or chimpanze or chimpanzees or chimps or cow or cows or "D melanogaster" or "dairy calf" or "dairy calves" or deer or dog or dogs or donkey or donkeys or drosophila or "Drosophila melanogaster" or duck or duckling or ducklings or ducks or equid or equids or equine or equines or feline or felines or ferret or ferrets or finch or finches or fish or flatworm or flatworms or fox or foxes or frog or frogs or "fruit flies" or "fruit fly" or "G mellonella" or "Galleria mellonella" or geese or gerbil or gerbils or goat or goats or goose or gorilla or gorillas or hamster or hamsters or hare or hares or heifer or heifers or horse or horses or insect or insects or jellyfish or kangaroo or kangaroos or kitten or kittens or lagomorph or lagomorphs or lamb or lambs or lemur or lemurs or llama or llamas or macaque or macaques or macaw or macaws or marmoset or marmosets or mice or minipig or minipigs or mink or minks or monkey or monkeys or mouse or mule or mules or nematode or nematodes or octopus or octopuses or orangutan or "orang-utan" or orangutans or "orang-utans" or ostrich or ostriches or oxen or parrot or parrots or pig or pigeon or pigeons or piglet or piglets or pigs or porcine or primate or primates or quail or rabbit or rabbits or rat or rats or reptile or reptiles or rodent or rodents or ruminant or ruminants or salmon or sheep or shrimp or slug or slugs or swine or tamarin or tamarins or toad or toads or trout or urchin or urchins or vole or voles or waxworm or waxworms or wildlife or worm or worms or xenopus or "zebra fish" or zebrafish) not (human or humans or patient or patients)).ti,ab,hw,kf.	11391556
22	19 not (20 or 21)	1088
23	limit 22 to (dissertation abstract or conference abstract or conference paper or conference proceedings or preprint or pre-print or editorial or erratum or note or addresses or autobiography or bibliography or biography or blogs or comment or	172

	dictionary or directory or interactive tutorial or interview or lectures or legal cases or legislation or news or newspaper article or overall or patient education handout or periodical index or portraits or published erratum or video-audio media or webcasts or conference review or clinical trial protocol or preprint or pre-print) [Limit not valid in APA PsycInfo,CCTR,CDSR,Embase,Ovid MEDLINE(R); records were retained]	
24	22 not 23	916
25	remove duplicates from 24	565

Scopus

1 TITLE-ABS-KEY(Acalculia OR agnosia* OR Agrammatism OR Agraphia* OR alogia* OR anepia* OR anomia* OR anomy OR anosognosia* OR aphasia OR aphasia* OR "dejerine lichtheim phenomenon" OR dysphasia* OR "Landau Kleffner" OR language OR "language impairment*" OR "language outcome*" OR "language recovery" OR "lichtheim sign" OR "lichtheims sign" OR linguistic OR logagnosia* OR logamnesia* OR logasthenia* OR "mesulam syndrome*" OR "mesulams syndrome*" OR naming OR "post-stroke aphasia" OR prosopagnosia* OR "semantic dementia*" OR "temporal variant frontotemporal dementia*" OR "temporal variant FTD" OR "temporal variant of frontotemporal dementia*" OR "temporal variant of FTD" OR tvFTD OR "Word Deaf*")

2 TITLE-ABS-KEY(((cerebral or brain or cerebrovascular* or "cerebral vascular*" or "cerebrum vascular*") W/3 (insult or insultus or accident* or "blood flow disturbance*" or infarct* or ischem* or ischaem*)) OR ((cerebrovascular* or "cerebral vascular*" or "cerebrum vascular*") W/3 (event* or disorder*)) OR apoplexia OR apoplexy OR "ischaemic seizure*" OR "ischemic seizure*" OR poststroke* OR "post-stroke*" OR stroke OR strokes)

3 TITLE-ABS-KEY("advanced diffusion imaging" OR "beyond-DTI imaging" OR "diffusion kurtosis imaging" OR "diffusion MRI" OR "diffusion MRIs" OR "diffusion tensor imaging" OR "diffusion tensor magnetic resonance imaging" OR "diffusion tensor mri" OR "diffusion tensor mris" OR "diffusion tractograph*" OR "diffusional spectral imaging" OR "diffusion-weighted imaging" OR "diffusion-weighted magnetic resonance imaging" OR "diffusion-weighted MRI" OR "diffusion-weighted MRIs" OR "dti mri" OR "dti mris" OR HARDI OR NODDI OR tractograph* OR "white matter tractography")

4 LANGUAGE(english)

5 1 and 2 and 3 and 4

6 TITLE-ABS-KEY(newborn* or neonat* or infant* or toddler* or child* or adolescent* or paediatric* or pediatric* or girl or girls or boy or boys or teen or teens or teenager* or preschooler* or "pre-schooler*" or preteen or preteens or "pre-teen" or "pre-teens" or youth or youths) AND NOT TITLE-ABS-KEY(adult OR adulthood OR adults OR centenarian* OR elderly OR geriatric* OR "middle age" OR "middle aged" OR nonagenarian* OR octogenarian* OR "old adult*" OR "old people" OR "old person*" OR "older adult*" OR "older people" OR "older person*" OR septuagenarian* OR Sextenarian* OR "very old")

7 5 and not 6

8 TITLE-ABS-KEY((alpaca OR alpacas OR amphibian OR amphibians OR animal OR animals OR antelope OR armadillo OR armadillos OR avian OR baboon OR baboons OR beagle OR beagles OR bee OR bees OR bird OR birds OR bison OR bovine OR buffalo OR buffaloes OR buffalos OR "c elegans" OR "Caenorhabditis elegans" OR camel OR camels OR canine OR canines OR carp OR cats OR cattle OR chick OR chicken OR chickens OR chicks OR chimp OR chimpanze OR chimpanzees OR chimps OR cow OR cows OR "D melanogaster" OR "dairy calf" OR "dairy calves" OR deer OR dog OR dogs OR donkey OR donkeys OR drosophila OR "Drosophila melanogaster" OR duck OR duckling OR ducklings OR ducks OR equid OR equids OR equine OR equines OR feline OR felines OR ferret OR ferrets OR finch OR finches OR fish OR flatworm OR flatworms OR fox OR foxes OR frog OR frogs OR "fruit flies" OR "fruit fly" OR "G mellonella" OR "Galleria mellonella" OR geese OR gerbil OR gerbils OR goat OR goats OR goose OR gorilla OR gorillas OR hamster OR hamsters OR hare OR hares OR heifer OR heifers OR horse OR horses OR insect OR insects OR jellyfish OR kangaroo OR kangaroos OR kitten OR kittens OR lagomorph OR lagomorphs OR lamb OR lambs OR llama OR llamas OR macaque OR macaques OR macaw OR macaws OR marmoset OR marmosets OR mice OR minipig OR minipigs OR mink OR minks OR

	monkey OR monkeys OR mouse OR mule OR mules OR nematode OR nematodes OR octopus OR octopuses OR orangutan OR "orang-utan" OR orangutans OR "orang-utans" OR oxen OR parrot OR parrots OR pig OR pigeon OR pigeons OR piglet OR piglets OR pigs OR porcine OR primate OR primates OR quail OR rabbit OR rabbits OR rat OR rats OR reptile OR reptiles OR rodent OR rodents OR ruminant OR ruminants OR salmon OR sheep OR shrimp OR slug OR slugs OR swine OR tamarin OR tamarins OR toad OR toads OR trout OR urchin OR urchins OR vole OR voles OR waxworm OR waxworms OR worm OR worms OR xenopus OR "zebra fish" OR zebrafish) AND NOT (human OR humans or patient or patients))
9	7 and not 8
10	DOCTYPE(ab) OR DOCTYPE(ed) OR DOCTYPE(bk) OR DOCTYPE(er) OR DOCTYPE(no) OR DOCTYPE(sh)
11	9 and not 10
12	INDEX(embase) OR INDEX(medline) OR PMID(0* OR 1* OR 2* OR 3* OR 4* OR 5* OR 6* OR 7* OR 8* OR 9*)
13	11 and not 12
