# Supplementary material for: Diffusion Tensor Imaging and Advanced Diffusion Imaging in Post-Stroke Aphasia Recovery
Source: Tomography. 2026 Feb 23;12(2):28. doi: 10.3390/tomography12020028 (PMC12944503; doi:10.3390/tomography12020028)
Supplement: Supplementary file 1 [file tomography-12-00028-s001.zip › Supplementary Tables S1-S5.pdf]

*Table 1 - Revised JBI critical appraisal tool for randomized controlled trials*

| STUDY ID | OUTCOME | RESULT | Q1 | Q2 | Q3 | Q4 | Q5 | Q6 | Q7 | Q8 | Q9 | Q10 | Q11 | Q12 | Q13 |
|---|---|---|---|---|---|---|---|---|---|---|---|---|---|---|---|
| Lin (2023) | microstructural integrity metrics | Time 1 | Y | Y | Y | Y | Y | Y | Y | Y | Y | Y | Y | Y | Y |
| | | Time 2 | | | | | | | | | | Y | Y | Y | |
| | language measures | Time 1 | | | | | | | Y | Y | Y | Y | Y | Y | |
| | | Time 2 | | | | | | | | | | Y | Y | Y | |
| Rosso (2014) | language measures | Time 1 | Y | U | Y | Y | U | Y | Y | Y | Y | Y | Y | Y | Y |
| | | Time 2 | | | | | | | | | | Y | Y | Y | |
| Sihvonen (2021) | microstructural integrity metrics | Time 1 | Y | U | Y | Y | U | Y | Y | Y | Y | Y | Y | Y | Y |
| | | Time 2 | | | | | | | | | | Y | Y | Y | |
| | language meaures | Time 1 | | | | | | | Y | Y | Y | Y | Y | Y | |
| | | Time 2 | | | | | | | | | | Y | Y | Y | |
| Sihvonen (2024) | microstructural integrity metrics | Time 1 | Y | U | Y | U | U | Y | Y | Y | Y | Y | Y | Y | Y |
| | | Time 2 | | | | | | | | | | U | U | Y | |
| | language meaures | Time 1 | | | | | | | Y | Y | Y | Y | Y | Y | |
| | | Time 2 | | | | | | | | | | U | U | Y | |
| Sihvonen (2022) | microstructural integrity metrics | Time 1 | Y | U | Y | Y | U | Y | Y | Y | Y | Y | Y | Y | Y |
| | | Time 2 | | | | | | | | | | Y | Y | Y | |
| | language meaures | Time 1 | | | | | | | Y | Y | Y | Y | Y | Y | |
| | | Time 2 | | | | | | | | | | Y | Y | Y | |
| Soliman (2021) | microstructural integrity metrics | Time 1 | Y | U | Y | Y | U | Y | U | Y | Y | Y | Y | Y | Y |
| | | Time 2 | | | | | | | | | | N | U | Y | |
| | language meaures | Time 1 | | | | | | | U | Y | Y | Y | Y | Y | |
| | | Time 2 | | | | | | | | | | Y | Y | Y | |
| Stockbridge (2024) | microstructural integrity metrics | Time 1 | Y | U | Y | U | Y | Y | Y | Y | Y | Y | Y | Y | Y |
| | | Time 2 | | | | | | | | | | U | U | Y | |
| | language meaures | Time 1 | | | | | | | Y | Y | Y | Y | Y | Y | |
| | | Time 2 | | | | | | | | | | Y | Y | Y | Y |
| Wang (2025) | FA change | Time 1 | Y | U | Y | U | U | Y | U | Y | Y | Y | Y | Y | |
| | | Time 2 | | | | | | | | | | U | U | Y | |

| | | | | | | | | | | | | | | | |
|---|---|---|---|---|---|---|---|---|---|---|---|---|---|---|---|
| | | Time 3 | | | | | | | | | | U | U | Y | |
| | language measures | Time 1 | | | | | | | U | Y | Y | Y | Y | Y | |
| | | Time 2 | | | | | | | | | | Y | Y | Y | |
| | | Time 3 | | | | | | | | | | Y | Y | Y | |
| Wilmskoetter (2022) | global network architecture | Time 1 | Y | U | Y | Y | U | Y | U | Y | Y | Y | Y | Y | Y |
| | | Time 2 | | | | | | | | | | U | U | Y | |
| | language measures | Time 1 | | | | | | | U | Y | Y | Y | Y | Y | |
| | | Time 2 | | | | | | | | | | Y | Y | Y | |

Y: Yes N: No U: Unclear N/A: Not applicable

Q1: Was true randomization used for assignment of participants to treatment groups?

Q2: Was allocation to treatment groups concealed?

Q3: Were treatment groups similar at the baseline?

Q4: Were participants blind to treatment assignment?

Q5: Were those delivering the treatment blind to treatment assignment?

Q6: Were treatment groups treated identically other than the intervention of interest?

Q7: Were outcome assessors blind to treatment assignment?

Q8: Were outcomes measured in the same way for treatment groups?

Q9: Were outcomes measured in a reliable way?

Q10: Was follow-up complete and, if not, were differences between groups in terms of their follow-up adequately described and analyzed?

Q11: Were participants analyzed in the groups to which they were randomized?

Q12: Was appropriate statistical analysis used?

Q13: Was the trial design appropriate and any deviations from the standard RCT design (individual randomization, parallel groups) accounted for in the conduct and analysis of the trial?

*Table 2 - Revised JBI critical appraisal tool for quasi-experimental studies*

| STUDY ID | OUTCOME | RESULT | Q1 | Q2 | Q3 | Q4 | Q5 | Q6 | Q7 | Q8 | Q9 |
|---|---|---|---|---|---|---|---|---|---|---|---|
| Allondorfer (2012) | FA change | Time 1 | Y | N | Y | Y | Y | Y | Y | Y | Y |
| | | Time 2 | | | | | | | | Y | Y |
| Bonilha (2016) | global network architecture | Time 1 | Y | N | Y | Y | Y | Y | Y | Y | Y |
| | | Time 2 | | | | | | | | Y | Y |
| | language meausures | Time 1 | | | | | Y | Y | Y | Y | Y |
| | | Time 2 | | | | | | | | Y | Y |
| Braun (2022) | microstructural integrity metrics | Time 1 | Y | N | Y | Y | Y | Y | Y | Y | Y |
| | | Time 2 | | | | | | | | Y | Y |
| | behavioral measures | Time 1 | | | | | Y | Y | Y | Y | Y |
| | | Time 2 | | | | | | | | Y | Y |
| Chang (2021) | microstructural integrity metrics | Time 1 | Y | N | Y | Y | Y | Y | Y | Y | Y |
| | | Time 2 | | | | | | | | Y | Y |
| | language meaures | Time 1 | | | | | Y | Y | Y | Y | Y |
| | | Time 2 | | | | | | | | Y | Y |
| Low (2024) | global network architecture | Time 1 | Y | Y | Y | Y | Y | Y | Y | Y | Y |
| | | Time 2 | | | | | | | | Y | Y |
| | language meaures | Time 1 | | | | | Y | Y | Y | Y | Y |
| | | Time 2 | | | | | | | | Y | Y |
| McKinnon (2017) | MK changes | Time 1 | Y | N | Y | Y | Y | Y | Y | Y | Y |
| | | Time 2 | | | | | | | | Y | Y |
| | language measures | Time 1 | | | | | Y | Y | Y | Y | Y |
| | | Time 2 | | | | | | | | Y | Y |
| Meier (2019) | language measures | Time 1 | Y | N | Y | Y | N | Y | Y | Y | Y |
| | | Time 2 | | | | | | | | Y | Y |
| Roth (2023) | language measures | Time 1 | Y | N | Y | Y | Y | Y | Y | Y | Y |
| | | Time 2 | | | | | | | | Y | Y |
| Wan (2014) | microstructural integrity metrics | Time 1 | Y | U | Y | Y | Y | U | Y | Y | Y |
| | | Time 2 | | | | | | | | Y | Y |

| | | | | | | | | | | | |
|---|---|---|---|---|---|---|---|---|---|---|---|
| | language measures | Time 1 | | | | | Y | U | Y | Y | Y |
| | | Time 2 | | | | | | | | Y | Y |
| Yu (2019) | language measures | Time 1 | Y | N | Y | Y | Y | Y | Y | Y | Y |
| | | Time 2 | | | | | | | | Y | Y |

Y: Yes N: No U: Unclear N/A: Not applicable

Q1: Is it clear in the study what is the “cause” and what is the “effect” (ie, there is no confusion about which variable comes first)?

Q2: Was there a control group?

Q3: Were participants included in any comparisons similar?

Q4: Were the participants included in any comparisons receiving similar treatment/care, other than the exposure or intervention of interest?

Q5: Were there multiple measurements of the outcome, both pre and post the intervention/exposure?

Q6: Were the outcomes of participants included in any comparisons measured in the same way?

Q7: Were outcomes measured in a reliable way?

Q8: Was follow-up complete and, if not, were differences between groups in terms of their follow-up adequately described and analyzed?

Q9: Was appropriate statistical analysis used?

*Table 3 – JBI Critical Appraisal Tool For Analytical Cross-sectional Studies*

| STUDY ID | Q1 | Q2 | Q3 | Q4 | Q5 | Q6 | Q7 | Q8 |
|---|---|---|---|---|---|---|---|---|
| Bonilha (2014) | Y | Y | Y | Y | Y | Y | Y | Y |
| Breier (2008) | Y | Y | Y | Y | Y | Y | Y | Y |
| Del Gaizo (2017) | Y | Y | Y | Y | Y | Y | Y | Y |
| Dickens (2021) | Y | Y | Y | Y | Y | Y | Y | Y |
| Dresang (2021) | Y | Y | Y | Y | Y | Y | Y | Y |
| Elmongui (2022) | Y | Y | Y | Y | U | U | Y | Y |
| Fan (2021) | Y | Y | Y | Y | Y | Y | Y | Y |
| Fridriksson (2018) | Y | Y | Y | Y | Y | Y | Y | Y |
| Geva (2015) | Y | Y | Y | Y | Y | Y | Y | Y |
| Gleichgerrcht (2015) | Y | Y | Y | Y | Y | Y | Y | Y |
| Gleichgerrcht (2016) | Y | Y | Y | Y | Y | U | Y | Y |
| Griffiths (2013) | Y | Y | Y | Y | Y | U | Y | Y |
| Han (2024) | Y | Y | Y | Y | Y | Y | Y | Y |
| Han (2016) | Y | Y | Y | Y | Y | Y | Y | Y |
| Harvey (2015) | Y | Y | Y | Y | Y | Y | Y | Y |
| Harvey (2013) | Y | Y | Y | Y | Y | Y | Y | Y |
| Hosomi (2009) | Y | Y | Y | Y | U | U | Y | Y |
| Hula (2020) | Y | Y | Y | Y | Y | Y | Y | Y |
| Ivanova (2016) | Y | Y | Y | Y | Y | Y | Y | Y |
| Ivanova (2021) | Y | Y | Y | Y | Y | Y | Y | Y |
| Keator (2021) | Y | Y | Y | Y | Y | Y | Y | Y |
| Keser (2023) | Y | Y | Y | Y | Y | Y | Y | Y |
| Keser (2021) | Y | Y | Y | Y | Y | Y | Y | Y |
| Kourtidou (2021) | Y | Y | Y | Y | U | U | Y | Y |
| Koyama (2016) | Y | Y | Y | Y | U | U | Y | Y |
| Kristinsson (2021) | Y | Y | Y | Y | Y | Y | Y | Y |
| Kyeong (2019) | Y | Y | Y | Y | Y | U | Y | Y |
| Lee (2021) | Y | Y | Y | Y | U | U | Y | Y |
| Lee (2018) | Y | Y | Y | Y | U | Y | Y | Y |

| | | | | | | | | |
|---|---|---|---|---|---|---|---|---|
| MartinezOeckel (2021) | Y | Y | Y | Y | Y | Y | Y | Y |
| Matchin (2024) | Y | Y | Y | Y | Y | Y | Y | Y |
| McCall (2022) | Y | Y | Y | Y | Y | Y | Y | Y |
| McKinnon (2018) | Y | Y | Y | Y | Y | Y | Y | Y |
| Medaglia (2022) | Y | Y | Y | Y | Y | Y | Y | Y |
| Noh (2021) | Y | Y | Y | Y | Y | Y | Y | Y |
| Olive (2023) | Y | Y | Y | Y | Y | Y | Y | Y |
| Olson (2025) | Y | Y | Y | Y | Y | Y | Y | Y |
| Papoutsi (2011) | Y | Y | Y | Y | Y | Y | Y | Y |
| Rolheiser (2011) | Y | Y | Y | Y | Y | Y | Y | Y |
| Rosso (2015) | Y | Y | Y | Y | Y | Y | Y | Y |
| Roth (2024) | Y | Y | Y | Y | Y | Y | Y | Y |
| Salvalaggio (2020) | Y | Y | Y | Y | Y | Y | Y | Y |
| Soliman (2023) | Y | Y | Y | Y | Y | Y | Y | Y |
| Tak (2013) | Y | Y | Y | Y | U | U | Y | Y |
| Wang (2020) | Y | Y | Y | Y | U | U | Y | Y |
| Xiao (2024) | Y | Y | Y | Y | Y | Y | Y | Y |
| Xing (2017) | Y | Y | Y | Y | Y | Y | Y | Y |
| Xing (2018) | Y | Y | Y | Y | Y | Y | Y | Y |
| Yang (2017) | Y | Y | Y | Y | U | U | Y | Y |
| Yurganov (2016) | Y | Y | Y | Y | U | U | Y | Y |
| Yu (2023) | Y | Y | Y | Y | Y | Y | Y | Y |
| Yu (2022) | Y | Y | Y | Y | Y | Y | Y | Y |
| Zhang (2018) | Y | Y | Y | Y | Y | Y | Y | Y |
| Zhang (2021b) | Y | Y | Y | Y | Y | Y | Y | Y |
| Zhoung (2022) | Y | Y | Y | Y | Y | Y | Y | Y |
| Zyryanov (2020) | Y | Y | Y | Y | Y | Y | Y | Y |

Y: Yes N: No U: Unclear N/A: Not applicable

Q1: Were the criteria for inclusion in the sample clearly defined?

Q2: Were the study subjects and the setting described in detail?

Q3: Was the exposure measured in a valid and reliable way?

Q4: Were objective, standard criteria used for measurement of the condition?

Q5: Were confounding factors identified?

Q6: Were strategies to deal with confounding factors stated?

Q7: Were the outcomes measured in a valid and reliable way?

Q8: Was appropriate statistical analysis used?

*Table 4 - JBI Critical Appraisal Tool For Analytical Cohort Studies*

| STUDY ID | Q1 | Q2 | Q3 | Q4 | Q5 | Q6 | Q7 | Q8 | Q9 | Q10 | Q11 |
|---|---|---|---|---|---|---|---|---|---|---|---|
| Agrawal (2024) | N/A | N/A | Y | Y | Y | Y | Y | Y | Y | U | Y |
| Bae (2022) | N/A | N/A | Y | Y | Y | Y | Y | Y | Y | U | Y |
| Blom-Smink (2020) | N/A | N/A | Y | Y | U | Y | Y | Y | Y | U | Y |
| Forkel (2018) | N/A | N/A | Y | Y | Y | Y | Y | Y | U | U | Y |
| Forkel (2014) | N/A | N/A | Y | Y | Y | Y | Y | Y | U | U | Y |
| Jang (2017) | N/A | N/A | Y | U | U | Y | Y | Y | Y | Y | Y |
| Keser (2020a) | N/A | N/A | Y | Y | Y | Y | Y | Y | U | Y | Y |
| Keser (2020b) | N/A | N/A | Y | Y | Y | Y | Y | Y | Y | Y | Y |
| Kim (2013) | N/A | N/A | Y | Y | U | Y | Y | Y | Y | Y | Y |
| Lee (2020) | N/A | N/A | Y | Y | Y | Y | Y | Y | U | U | Y |
| Leo (2019) | Y | Y | Y | Y | Y | Y | Y | Y | Y | Y | Y |
| Moulton (2019) | N/A | N/A | Y | Y | Y | Y | Y | Y | U | U | Y |
| Osa Garcia (2024) | N/A | N/A | Y | Y | Y | Y | Y | Y | N | N | Y |
| Schevenels (2022) | N/A | N/A | Y | Y | Y | Y | Y | Y | Y | Y | Y |
| Sihvonen (2022a) | N/A | N/A | Y | Y | Y | Y | Y | Y | Y | Y | Y |
| Sihvonen (2023) | N/A | N/A | Y | Y | Y | Y | Y | Y | Y | U | Y |
| Zhang (2021) | N/A | N/A | Y | Y | Y | Y | Y | Y | N | N | Y |

Y: Yes N: No U: Unclear N/A: Not applicable

Q1: Were the two groups similar and recruited from the same population?

Q2: Were the exposures measured similarly to assign people to both exposed and unexposed groups?

Q3: Was the exposure measured in a valid and reliable way?

Q4: Were confounding factors identified?

Q5: Were strategies to deal with confounding factors stated?

Q6: Were the groups/participants free of the outcome at the start of the study (or at the moment of exposure)?

Q7: Were the outcomes measured in a valid and reliable way?

Q8: Was the follow up time reported and sufficient to be long enough for outcomes to occur?

Q9: Was follow up complete, and if not, were the reasons to loss to follow up described and explored?

Q10: Were strategies to address incomplete follow up utilized?

Q11: Was appropriate statistical analysis used?

*Table 5 - JBI Critical Appraisal Tool For Case Series*

| Study ID | Q1 | Q2 | Q3 | Q4 | Q5 | Q6 | Q7 | Q8 | Q9 | Q10 |
|---|---|---|---|---|---|---|---|---|---|---|
| Guo (2007) | Y | Y | Y | Y | Y | Y | Y | Y | Y | Y |
| Kim (2011) | Y | Y | Y | Y | Y | Y | Y | U | Y | U |

*Y: Yes N: No U: Unclear N/A: Not applicable*

Q1: Were there clear criteria for inclusion in the case series?

Q2: Was the condition measured in a standard, reliable way for all participants included in the case series?

Q3: Were valid methods used for identification of the condition for all participants included in the case series?

Q4: Did the case series have consecutive inclusion of participants?

Q5: Did the case series have complete inclusion of participants?

Q6: Was there clear reporting of the demographics of the participants in the study?

Q7: Was there clear reporting of clinical information of the participants?

Q8: Were the outcomes or follow up results of cases clearly reported?

Q9: Was there clear reporting of the presenting site(s)/clinic(s) demographic information?

Q10: Was statistical analysis appropriate?
